# Case report of a patient with an intraosseous meningioma presenting as possible metastasis from prostate cancer: Diagnostic dilemma and review of literature

**DOI:** 10.1016/j.radcr.2024.07.041

**Published:** 2024-08-09

**Authors:** Prateek Mehra, Daniel Tesolin, Julia Malone, Gerard Jansen, John Sinclair, Shawn Malone

**Affiliations:** a The Ottawa Hospital Cancer Centre, Ottawa Hospital Regional Cancer Program, Ottawa, Canada; b The Ottawa Hospital Department of Pathology and Laboratory Medicine, Division of Anatomical Pathology, Ottawa Hospital General Campus, Ottawa, Canada; c The Ottawa Hospital Neurosciences Clinic, Ottawa Hospital Civic Campus, Ottawa, Canada

**Keywords:** Carcinoma prostate, Intraosseous meningioma, Oligometastatic

## Abstract

Intraosseous meningiomas are a rare subtype of meningiomas representing approximately 2% of all cases. They can confound a diagnosis of other bone lesions including metastatic tumors. We present a case of a patient with prostate cancer who on staging workup was suspected to have a skull metastasis. Both bone scan and CT Head demonstrated a lesion in the right frontal calvarium. Surgical resection and pathology revealed an intraosseous meningioma. The patient was restaged as having localized prostate cancer and the was offered curative treatment for his malignancy. The case highlights the importance of obtaining tissue diagnosis in cases of radiographic isolated oligometastatic disease in patients with a known primary malignancy.

## Introduction

Meningiomas arise from the pia arachnoid layer of the meninges. Most meningiomas are intradural and extramedullary in location. Intraosseous meningiomas are a rare subtype of meningioma which arise from the bone itself [1]. A majority of them are found in the skull convexity, mostly around skull sutures in the periorbital or frontoparietal regions [2]. We report on a patient with prostate cancer with a prostate specific antigen (PSA) greater than 20 who had a solitary sclerotic bone lesion in keeping with an isolated bone metastasis. Following surgical removal of the bone lesion it was determined that the sclerotic lesion was an intraosseous meningioma and the patient had localized prostate cancer. As a result, the patient was offered curative treatment of his prostate cancer. The case highlights the importance of obtaining tissue confirmation in cases with presumed solitary metastases.

## Case presentation

A 65-year-old male with a long-standing history of low-risk prostate cancer was seen for staging work-up after being on active surveillance for a low-risk prostate cancer. The patient was healthy with no major medical co-morbidities. He had a prior surgical history of cholecystectomy. Initially, he had a prostate biopsy in 2013, which revealed a high-grade Prostate Intraepithelial Neoplasia (PIN) in 2 of 10 cores. At the time his PSA was elevated at 6.2. Repeat prostate biopsy in 2015 demonstrated 1 of 14 cores positive for a Gleason 3+3 adenocarcinoma. Digital rectal examination revealed an enlarged prostate with no palpable tumor, and he was staged T1c disease. He was considered low risk disease and was offered active surveillance by his urologist. He continued to have repeat biopsies every 2 years while on active surveillance over an 8-year interval. Repeat biopsies were negative for malignancy during this time frame.

In 2022 his PSA rose to 26.6 ug/L. A 3 Tesla multiparametric MRI prostate was performed. Two PI-RADS 4 lesions were identified including a 12 mm lesion in the right transition zone and 6 mm in the right peripheral zone, both contained within the prostate. Repeat biopsy revealed Gleason 3+3 adenocarcinoma of the prostate in 2 of 15 cores. There was no evidence of perineural invasion. Ultrasound estimated the prostate volume to be 86 ml. The patient's clinical course was complicated by post biopsy urinary retention requiring a temporary Foley catheter insertion. In addition, the patient developed a left lower limb deep vein thrombosis and bilateral pulmonary emboli. The patient was treated with the antithrombotic apixaban. It was unclear if the thrombosis was caused by his prostate cancer or unprovoked. These events delayed management of his cancer by several months.

The patient recovered from his pulmonary emboli and remained otherwise clinically well with no symptoms to suggest metastatic disease. His only symptoms were moderate lower urinary tract obstructive symptoms. The patient was eligible for either radical prostatectomy or radical radiotherapy. Because of his PSA level, staging bone scan and CT abdomen and pelvis were ordered. PSMA PET scans are not currently funded in Ontario for routine staging of prostate cancer.

**Fig. 1:**
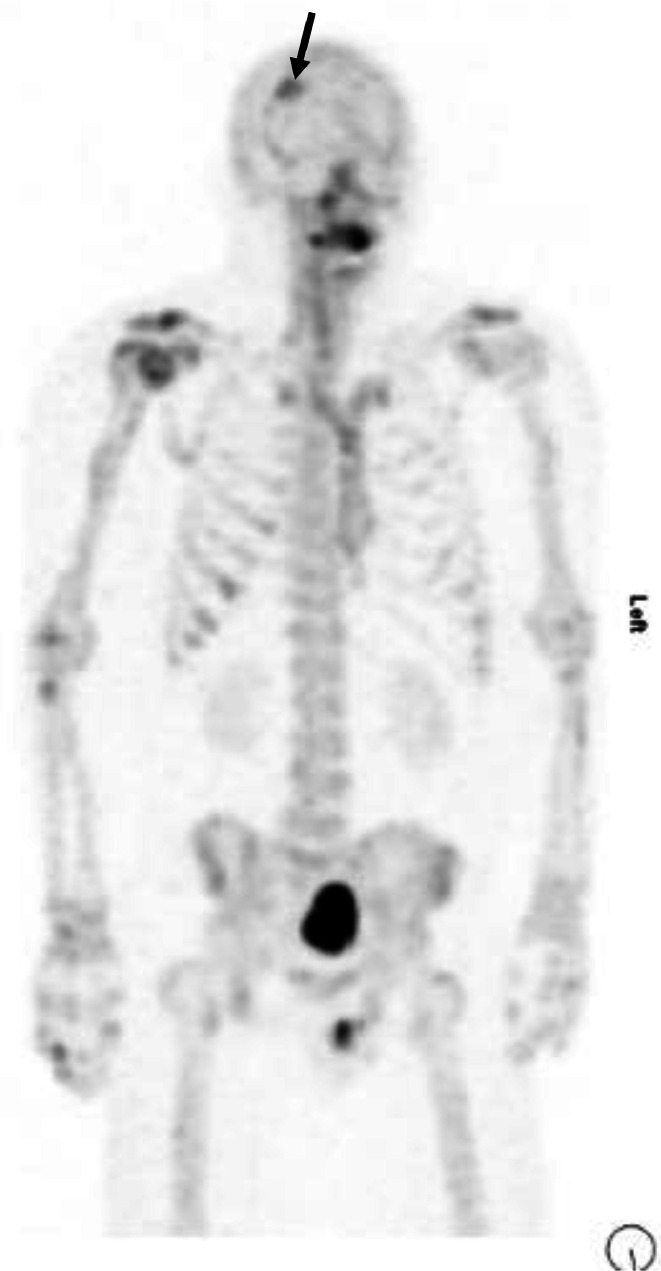
The suspected skull metastasis as seen on a 99m-Tc bone scan.

**Fig. 2:**
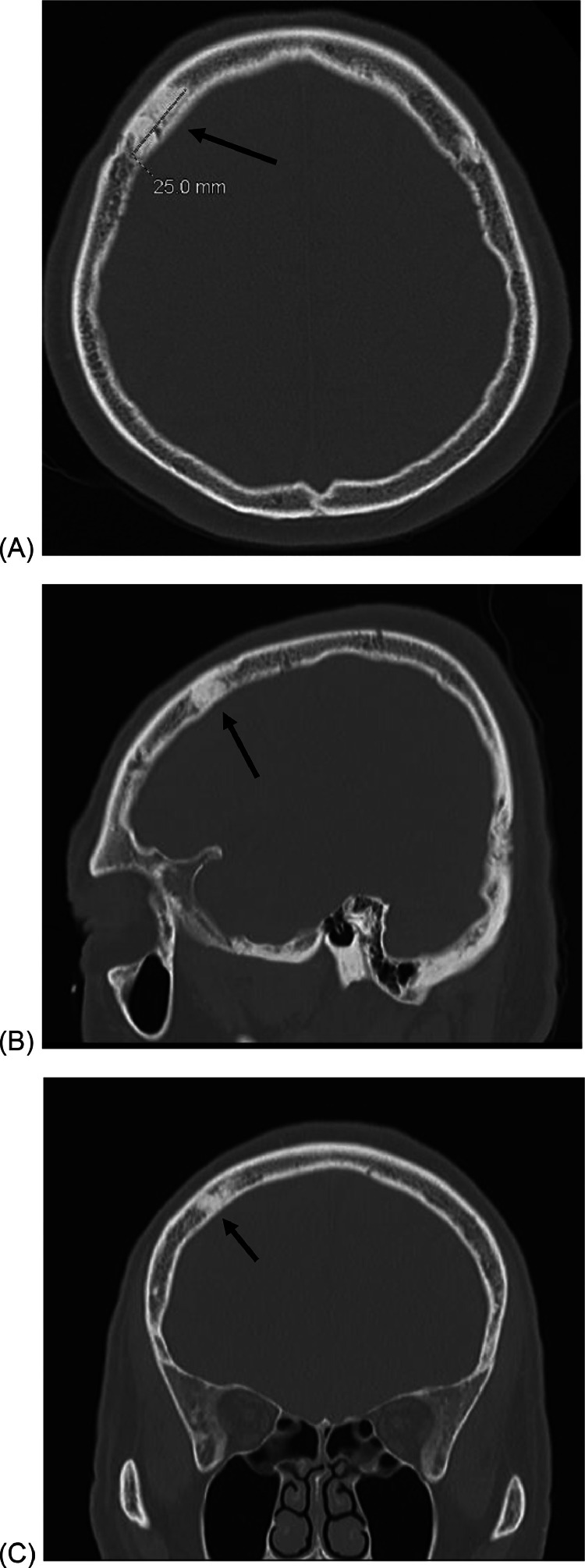
(A) Axial, (B) Sagittal and (C) Coronal CT scan of the suspected skull metastasis.

CT chest, abdomen and pelvis did not reveal evidence of bone or nodal metastases. Bone scan revealed moderate increased focal uptake in the right frontal calvarium, thought to be consistent with metastatic disease. CT head demonstrating a 2.5 cm sclerotic lesion in the right frontal bone. The CT abnormality corresponded to the area of uptake on the bone scan and was suspicious for a sclerotic metastasis (Figs. 1 and 2).

The patient was seen in consultation by Radiation Oncology. Because the skull abnormality was an isolated radiologic site of presumed metastasis, the oncologist recommended obtaining tissue diagnosis of the skull lesion to confirm if it was metastatic or a different pathology.

**Fig. 3:**
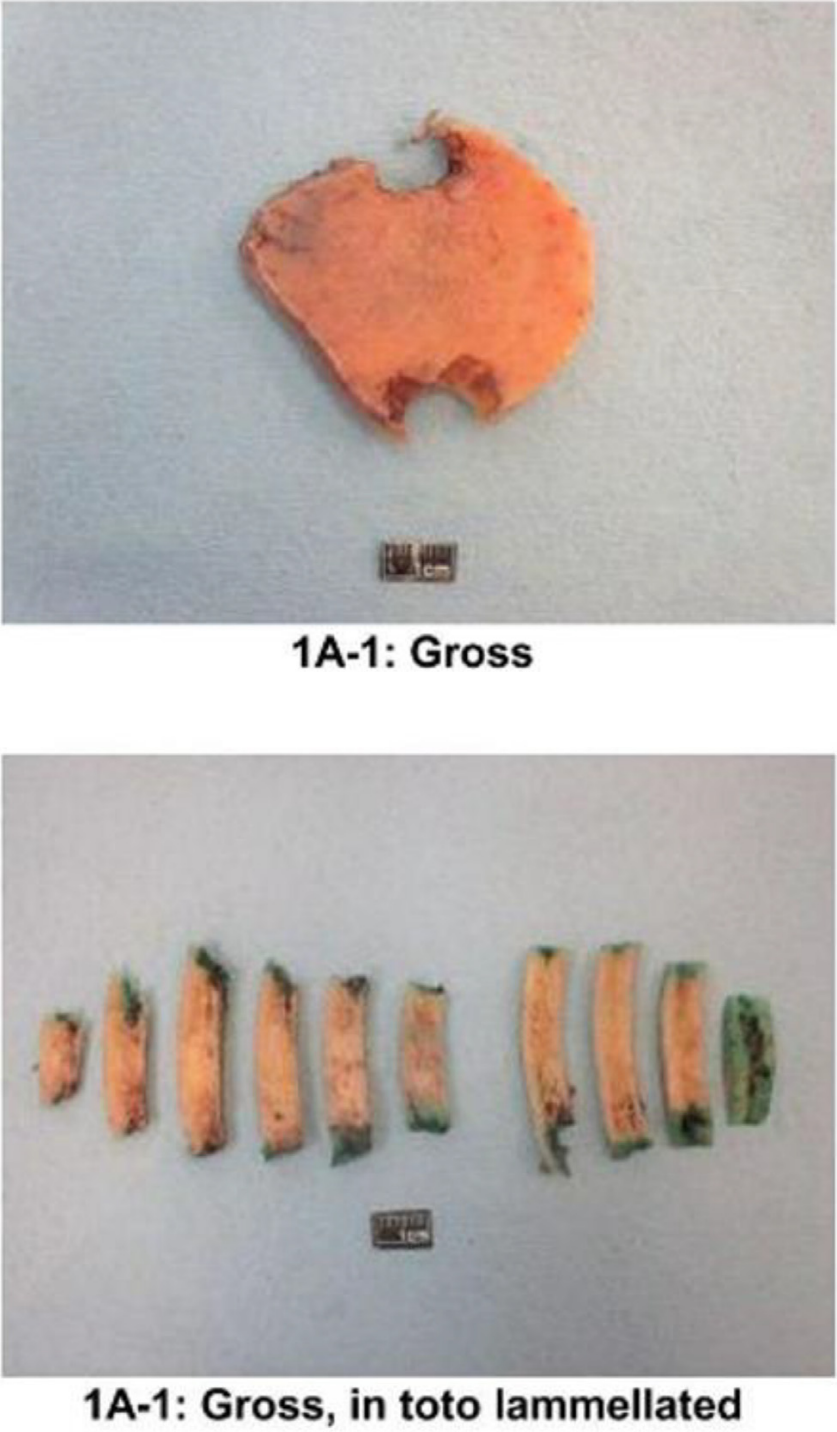
Gross features of the surgical specimen post gross total-resection.

**Fig. 4:**
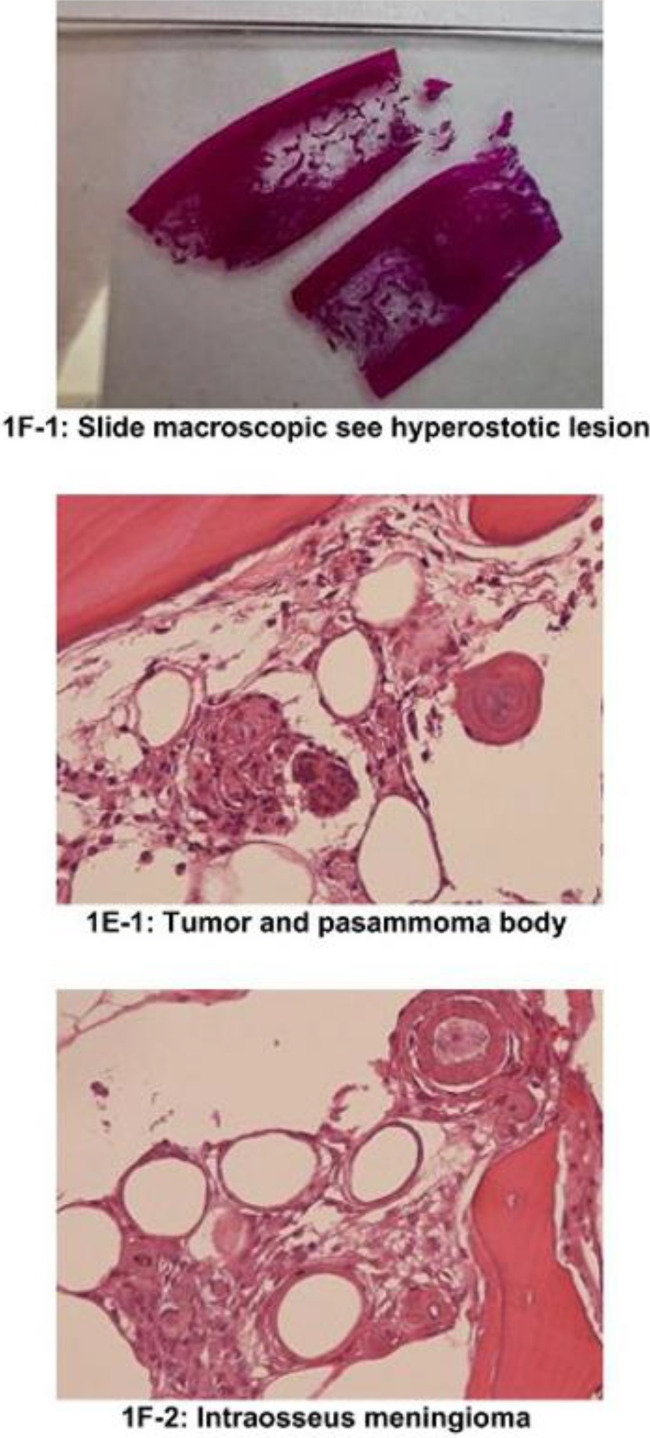
Microscopic features of the intraosseous meningioma.

**Fig. 5:**
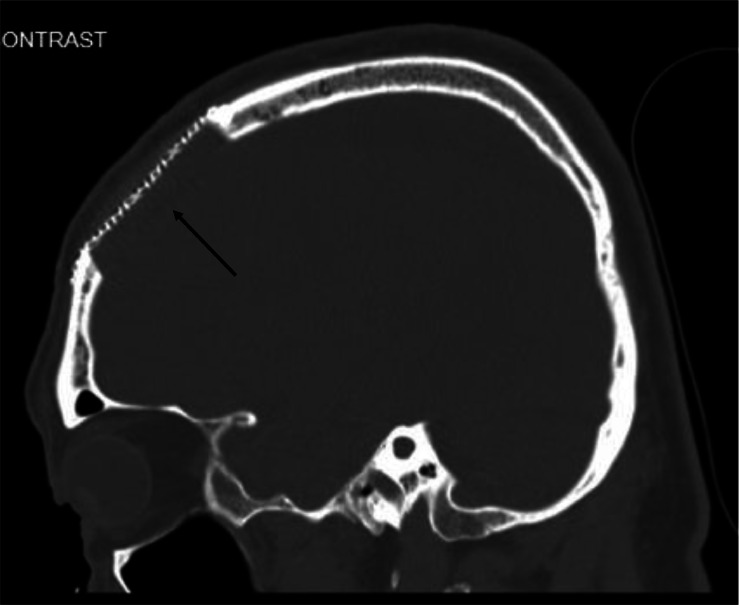
Post-op CT showing titanium plate over the cranial defect.

The patient was referred to neurosurgery and they performed a gross total resection of the frontal bone lesion. During the surgery, the tumor, skull and dura were removed en bloc and intraoperative analysis deemed it to be a gross total resection with negative margins. Surgical pathology revealed an unifocal intraosseous meningioma WHO grade 1 with hyperostosis (Figs. 3 and 4). Immunohistochemistry revealed the lesion was PSA negative, androgen receptor negative, EMA +2 positive, and Vimentin +3 positive, consistent with meningioma. On final pathology, resection margins were clear confirming a gross total resection. Postoperative imaging confirmed a gross total excision (Fig. 5). His postoperative course was complicated by a subdural hematoma. Within weeks he fully recovered from surgery with no postoperative deficits. No intervention was needed for the subdural hematoma.

The neurosurgery and oncology teams deemed the intraosseous meningioma had received definitive treatment with gross total resection and did not require adjuvant treatment. The patient will undergo regular imaging surveillance to for the meningioma to rule out recurrence in the future. In terms of his prostate cancer, he was offered the option of surgery or radical radiotherapy. The patient opted for radical external beam radiation.

## Discussion

Primary intraosseous meningiomas (PIMs) are rare tumors that arise from the bone. The majority of these lesions are osteoblastic and appear sclerotic on CT scan. The imaging resulted in a diagnostic dilemma in our patient with prostate cancer who had a PSA above 20. Less than 20% of PIMs are osteolytic [2]. An analysis by Liu et al revealed that intraosseous meningiomas tend to be located in skull convexities, paranasal sinuses, and middle ear [3]. The origin of PIMs remains uncertain and multiple competing theories having been proposed. One theory proposes misplacement of mesenchymal stem cells [4] while other states that arachnoid cap cells get trapped in cranial sutures during cranial molding at birth or during embryogenesis [5,6]. Trauma has also been proposed as a possible cause, with dura getting trapped in the bone and leading to a meningioma later in life [7].

Epidemiologically, these meningiomas have a bimodal age of presentation—one peak during second decade of life and the other during the fifth to seventh decades. When occurring in the skull, PIMs are usually seen in frontoparietal or periorbital regions [2]. They are usually painless and firm on palpation. The symptoms are dependent on the location of the lesion, i.e. which lobe of the brain they are compressing. However, neurological signs are very uncommon with occurrence of raised intracranial pressure in only 1.5% [3]. Temporal bone invasion can lead to auditory deficits. If they are found in the nasal cavity or in the paranasal sinuses, they can present with epistaxis or nasal obstruction.

Radiologically, PIMs are evaluated with CT and MRI [8]. CT helps in delineating the bony extent of the lesion (inner and outer tables and diploe). It can show the lesions as hyperostotic (most common presentation), osteolytic (second most common) or mixed [3]. MRI allows for evaluation of extradural extension and soft tissue involvement. PIMs appear hypointense on T1 imaging, hyperintense on T2 and heterogeneous on FLAIR sequences. The differential diagnosis, especially in a case such as our patient includes metastasis, fibrous dysplasia, osteoma, and Paget's disease.

Fibrous dysplasia tends to stop growing after puberty whereas PIMs continue growing independent of patient age. Fibrous dysplasia displays a smooth inner table, whereas it tends to be irregular in PIMs. When compared to osteomas, PIMs display homogeneous enhancement post contrast injection, however osteomas are nonenhancing. Paget disease of the skull shows a “cotton-wool” appearance, a characteristic not seen with PIMs.

Lang et al classified PIMs according to the lesion location [9]. Purely extra-calvarial tumors with no bony attachment are classified as Type 1, purely calvarial are Type 2. Type 2 lesions are divided into subtypes B and C, with skull base lesions being subtype B and skull convexity lesions being subtype C respectively. There is no subtype A. Calvarial tumors with extra-calvarial extension are Type 3 with the same subtypes B and C as Type 2. Our patient was classified as a Type 2C tumour. The treatment of choice for PIMs remains wide local excision [10,11]. On histopathology intraosseous meningiomas are graded as WHO Grade 1, 2 or 3 in the same way as intradural meningiomas. Since the present case was a Grade 1 meningioma with negative surgical margins, the neurosurgery and oncology teams decided that adjuvant treatment was not required. The patient will undergo serial clinical and imaging follow-up to rule out recurrence.

The sclerotic nature of the intraosseous meningioma in our case made it a diagnostic dilemma as our patient had prostate cancer and significantly elevated PSA. Like many metastatic prostate cancers the meningioma was osteoblastic in nature. Most intraosseous meningiomas are sclerotic on imaging. Lytic lesions occurring in less than 20% of cases [2]. The meningioma in this patient was a WHO Grade I, which is also the predominant type of PIM [2]. Recurrence is not uncommon with intraosseous meningioma, with rates of approximately 20 % found in the literature [9] While most PIMs have benign features, 26% of osteolytic intraosseous meningiomas are classified as WHO Grade II or III with a higher propensity for recurrence.

There have been other cases of clinical conditions which have confounded clinicians when staging carcinoma prostate. One case report from Canada discussed the metastasis of prostate cancer to a pre-existing meningioma in the patient [12]. The patient developed right orbital apex syndrome; a biopsy of the mass revealed a meningioma with metastasis of the patient's prostate cancer. A collection of cases from Chile looked at the pathological findings of meningeal masses in patients with malignancies. They were initially presumed to be meningiomas but on pathologic evaluation, were found to be thyroid, prostate or breast adenocarcinomas or hypernephromas [13]. Both of these papers also emphasize the need of pathologic evaluation of any suspected metastases, rather than just relying on radiologic findings.

There are multiple radiologic indicators that point towards metastatic disease versus a meningioma. Metastatic disease typically presents with destructive changes in the bone whereas meningiomas do not cause such changes but there can be reactive sclerotic changes in the bone abutting the meningioma. Intralesional necrosis and rapid growth over serial imaging are much more likely to indicate metastases as compared to meningiomas. Intralesional enhancement tends to be homogeneous in meningiomas but can be patchy enhancement in metastatic lesions.

## Conclusion

The case illustrates the importance of obtaining pathology to confirm diagnosis in management of oncology patients. While staging bone scan raised the suspicion of oligometastatic prostate cancer, it was only through surgery and histology that the patient was re-staged as having a curable organ-confined malignancy. The pathology findings had significant clinical implications for both the prognosis and management of the patients prostate cancer. The case highlights the need for both radiologic and pathologic findings to be correlated with each other before a final management decision is taken.

## Patient consent

We certify that written informed consent was obtained from the patient for the publishing of this case report.
